# Evaluating passive physiological data collection during Spravato treatment

**DOI:** 10.3389/fdgth.2023.1281529

**Published:** 2023-11-29

**Authors:** Todd M. Solomon, Matus Hajduk, Martin Majernik, Jamileh Jemison, Alexander Deschamps, Jenna Scoggins, Adam Kolar, Miguel Amável Pinheiro, Peter Dubec, Ondrej Skala, Owen Muir, Amanda Tinkelman, Daniel R. Karlin, Robert Barrow

**Affiliations:** ^1^ Global Clinical Development, Mind Medicine (MindMed), Inc., New York, NY, United States; ^2^ Brooklyn Minds Psychiatry & Curated Mental Health, New York, NY, United States; ^3^ Department of Psychiatry, Tufts University School of Medicine, Boston, MA, United States

**Keywords:** passive monitoring, spravato, esketamine, mental health, depression, psychedelic, dissociative, hallucinogen

## Abstract

Spravato and other drugs with consciousness-altering effects show significant promise for treating various mental health disorders. However, the effects of these treatments necessitate a substantial degree of patient monitoring which can be burdensome to healthcare providers and may make these treatments less accessible for prospective patients. Continuous passive monitoring via digital devices may be useful in reducing this burden. This proof-of-concept study tested the MindMed Session Monitoring System™ (MSMS™), a continuous passive monitoring system intended for use during treatment sessions involving pharmaceutical products with consciousness-altering effects. Participants completed 129 Spravato sessions with MSMS at an outpatient psychiatry clinic specializing in Spravato treatment. Results indicated high rates of data quality and self-reported usability among participants and health care providers (HCPs). These findings demonstrate the potential for systems such as MSMS to be used in consciousness-altering treatment sessions to assist with patient monitoring.

## 1. Introduction

The global rate of mental health disorders has increased significantly in the past several years, in part due to the effects of the COVID-19 pandemic. Recent data has indicated that the global prevalence of depressive and anxiety disorders increased by over 25% during 2020 (1). In the United States, the CDC estimates almost one-third of adults have significant anxiety or depression symptoms (2). A 2021 survey of psychologists conducted by the American Psychological Association (APA) indicated that 62% of respondents had an increase in the number of referrals since the start of the pandemic, with patients seeking treatment for anxiety, depression, and trauma-based disorders having the highest demand (3). Despite this continually increasing prevalence of mental health disorders, there remains a dearth of qualified treatment providers and efficacious pharmacological treatments.

One of the innovations in psychopharmacology in recent history was the discovery of the antidepressant effects of ketamine. Ketamine is an antagonist of the N-methyl-D-aspartate (NMDA) receptor and targets the excitatory amino acid neurotransmitter glutamate. It is well established to cause consciousness-altering effects such as dissociation and sedation, historically having been used as an anesthetic (4). In the 1990s and early 2000s, low doses of ketamine were found to alleviate depressive symptoms (5), which spurred considerable research into its rapid onset, but often transient, antidepressant effects (6). In 2019, the United States Food and Drug Administration (FDA) approved the S-enantiomer form of ketamine, esketamine (brand name Spravato), for use in patients with Treatment-Resistant Depression (TRD) and Major Depressive Disorder (MDD) with suicidal ideation. Spravato's efficacy has been demonstrated in numerous follow-on studies in a variety of populations (5, 7–13). While Spravato's efficacy and the need for new mental health treatments has driven significant growth, a major limitation to more rapid and accessible uptake is the monitoring requirements necessary during and after Spravato dosing as a part of its FDA-mandated Risk Evaluation and Mitigation Strategy (REMS) program (14, 15).

The Food and Drug Administration Amendments Act of 2007 provided the FDA the authority to require a REMS for certain drugs to ensure their benefits outweighed their associated safety risks (16). REMS requirements go beyond medication labeling, focusing on specific risks associated with the medication and mandating action to reduce the severity or frequency of those risks. As part of its approval, Spravato has an FDA-mandated REMS to mitigate its potential for abuse and misuse as well as potential adverse effects, including physiological changes, dissociation, and sedation. The Spravato REMS program involves training and certification for Spravato providers as well as direct observation and monitoring of patients undergoing Spravato treatment by a healthcare provider for a minimum of two hours after administration (17). Additionally, in order for a patient to be released from their treatment session, the REMS mandates that healthcare providers confirm a patient's blood pressure is in an “acceptable range” and that feelings or symptoms of dissociation have resolved (18).

There are other drugs with consciousness-altering effects currently in development for treating mental health disorders, such as psychedelics, which bind to 5HT2A serotonin receptors, and MDMA, which increases levels of serotonin, dopamine, and noradrenaline in the brain (19, 20). While the literature indicates these drugs have a strong potential to be effective treatments, questions remain regarding how to safely and efficiently administer these treatments at the scale needed to address the continued mental health crisis (21). Much like with the use of Spravato, the pharmacodynamic effects associated with many of the consciousness-altering molecules currently under development typically require patients to be continuously supervised and monitored for both physiological and psychological changes by one or more individuals while undergoing treatment (22). As several proprietary formulations of these consciousness-altering compounds are progressing through late-stage clinical trials (23), approval by regulatory agencies may be conditional on implementing a REMS program similar to Spravato (24). Although this ensures patient safety while under the effects of drugs that have consciousness-altering properties, the infrastructure needed for this level of monitoring is a potential barrier to their wide-scale adoption and use to improve treatment outcomes in mental health.

Continuous passive monitoring could help address issues in the implementation and regulation of consciousness-altering treatments. The use of passive monitoring technologies has had a prominent role in the improvement of healthcare outcomes in the last several decades, from technologies developed to monitor patients' vitals during general anesthesia (25), neurologic activity during sleep (26), or passive monitoring of glucose levels in diabetes (27). The technology has continued to evolve, and sensors have become smaller, more accessible, and easier to deploy. This evolution has led to rapid growth in medical grade software and technology that can be deployed on consumer-facing products. It has allowed for the development of such technologies such as the detection of atrial fibrillation through wrist-based heart rate monitoring (28) or detection of motor symptoms associated with early Parkinson's disease based on mobile phone accelerometer data (29). Passive monitoring via consumer-oriented devices such as smartphones and wearables has also started to be utilized as a means of tracking symptoms associated with mental health disorders such as depression and anxiety (30–32). While these technologies are already widely used in many medical specialties, they have yet to be deployed in a setting where Spravato, psychedelics, or MDMA are being administered.

Continuous passive monitoring may be useful during consciousness-altering treatments to gather and relay clinical and physiological data from patients in a way that alleviates the burden on both the health care provider (HCP) and the patient. To explore the use of this technology during consciousness-altering treatments, Mind Medicine Inc. (MindMed) conducted a proof-of-concept study in a mental health clinic specializing in facilitating Spravato treatment. MindMed is a clinical-stage biopharmaceutical company developing medicines for brain health disorders and aligned digital tools. MSMS is a proprietary software platform to be used by HCPs for continuous passive monitoring during therapy sessions involving pharmaceutical products with consciousness-altering effects. The objectives of the present study (MSMS-001) were: (1) to determine if MSMS can reliably and passively collect physiological data in a clinical setting during Spravato sessions, (2) to assess the quality of the collected data, and (3) to examine the acceptability of using MSMS to collect data from the point of view of both patients and practitioners. Results from this study will provide insight into the feasibility of using continuous passive monitoring via MSMS during treatment sessions involving consciousness-altering compounds.

## 2. Methods

### 2.1. Measures/devices

MSMS comprises a smartphone, smartwatch, instructions for use, and a user-facing mobile application. MSMS is designed to collect heart rate, accelerometer, gyroscope, compass, motion, audio, distance, steps, activity, and pedometer data. MSMS collects this data because of the physical side effects of Spravato, which can include increased blood pressure, dizziness, and disorientation (18). Additionally, collecting this broad range of data can allow us to explore potential biomarkers or indications that can give an HCP insight into a patient's safety and clinical progress during a Spravato session. For example, recent research has demonstrated the potential for the acoustic characteristics of speech to serve as biomarkers for depression and cognitive functioning (33, 34). As this is a proof-of-concept study, the potential for MSMS to accurately measure effects of Spravato or use biomarkers to detect clinical changes was not assessed. However, demonstrating the feasibility of MSMS to collect physiological data in a real-world setting is an important first step in building a device with such capabilities. We tested the feasibility and usability of MSMS in an IRB-approved study at a mental health clinic with an active Spravato treatment program.

### 2.2. Study visits

Clinic staff members pre-screened patients based on their health records and the clinical judgment of the study doctor. Interested patients provided informed consent for collecting physiological data via MSMS for up to eight Spravato sessions that were part of their existing treatment plan. Participants also consented to share their demographics, medical history, and the results of clinical measures collected during their treatment sessions. For any session, participants could choose not to have it recorded via MSMS while remaining enrolled in the study. Before dose administration, HCPs started recording on the devices via MSMS, placed the smartwatch on the participant's dominant wrist, and gave the smartphone to participants to place in their pocket or somewhere nearby, depending on patient preference.

All REMS protocols and Spravato treatment guidelines were followed during study sessions (18). The frequency of treatment sessions was based on each participant's pre-existing plan of care, with a mean of 11.6 days between treatment sessions across participants. In addition to passive monitoring done by MSMS, participants were monitored by HCPs throughout their treatment sessions. HCPs collected participant vitals (blood pressure and heart rate) at roughly 40- and 120-min post-dose. At 120 min post-dose, HCPs also performed the Clinician Administered Dissociative States Scale (CADSS) (35) and the Modified Observer's Assessment of Alertness/Sedation (MOASS) (36) to assess participant's levels of dissociation and sedation during and after the session. If the participant remained dissociated, sedated, or had abnormal vital signs 120 min post-dose, the participant was kept under observation and reassessed at 10-min intervals until the symptoms resolved. When the participant met the release criteria, the HCP ended passive recording and collected the MSMS devices from the participant.

**Figure 1:**
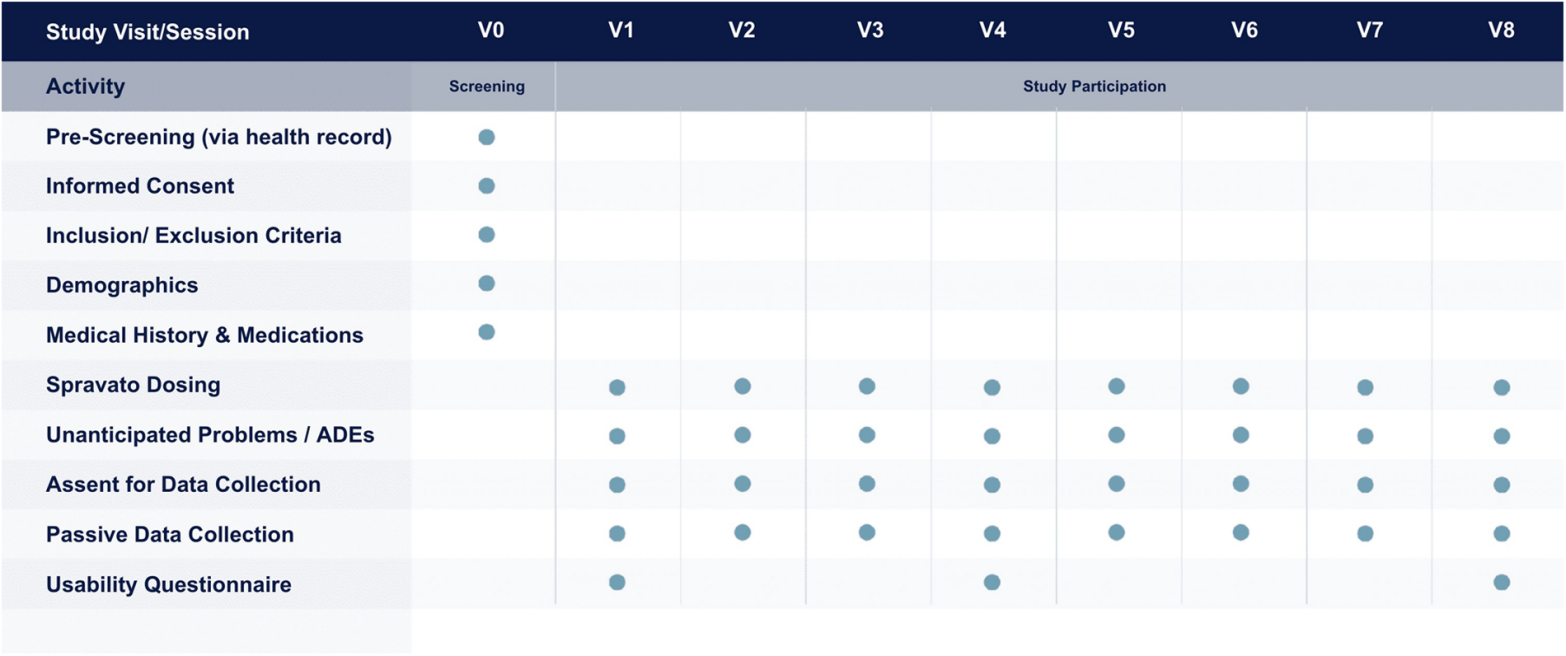
Study design and activities, including screening, per each participant visit.

After sessions 1, 4, and 8 during the study, participants and HCPs could complete usability surveys about their experience using MSMS while undergoing treatment. Separate surveys were given to participants and HCPs in order to gain insight into their respective experiences with MSMS. Figure 1 illustrates the study design and activities per visit.

A system was established to record and notify the study's Institutional Review Board (IRB) of any Serious Adverse Events (SAEs) and Unanticipated Device Problems (UDPs). No instances of SAEs or UDPs occurred.

### 2.3. Study population

We conducted this study in accordance with guidelines on human subjects research and approved by the Advara Institutional Review Board (#Pro00058933). Participants were recruited among patients receiving Spravato treatment at the study clinic.

Inclusion criteria were (1) 18 years of age or older at the time of enrollment; (2) currently receiving outpatient treatment through the research site; (3) willing to provide informed consent; (4) a clinical diagnosis appropriate for the treatment with Spravato in the judgment of the investigator; (5) willing to share passive data; (6) willing to share demographics, mental health history, and clinical data with the research sponsor. Exclusion criteria were (1) active in another clinical research study; (2) considered unsuitable for participation in the judgment of the investigator due to serious or unstable medical illnesses.

### 2.4. Data quality

#### 2.4.1. Data collection in a clinical setting

Determining if MSMS can reliably collect data in a clinical setting is a necessary step in establishing feasibility. Real-world environments such as outpatient psychiatry clinics may not always be optimal for passive data collection due to factors such as incompatible clinical workflows or poor internet connection. The study sought to determine to what extent the MSMS can capture all designated data types. This includes accelerometer, gyroscope, compass, motion, audio, activity, and pedometer data from the smartphone; heart rate, watch accelerometer, and watch motion from the smartwatch; and distance and steps data composited from both the smartwatch and smartphone.

#### 2.4.2. Data corruption

The study sought to examine if the values of the data collected were reasonable both in terms of absolute value and rate of change. For example, if we received data that indicated rapid, clinically unrealistic changes in heart rate or motion within a short period of time, those data are likely corrupted. We therefore analyzed the data values and rate of change to confirm they fall within specific predefined ranges for each data type. As this is a proof-of-concept study, we defined these ranges based on what we deemed logically reasonable given each data type and the nature of Spravato sessions. Future research in this field may refine and adjust these values to be more precise. Supplementary Table S1 displays the precise predefined ranges used to determine corruption rates for all data types collected in this study.

#### 2.4.3. Data coverage

Another essential component of data quality is ensuring that data is collected across the entirety of a Spravato session, rather than just a few data points collected at limited points in time. We measured data coverage rates by obtaining the start and end time of a session via clinical records and matching it to the passive data captured by MSMS to ensure they line up. We then further analyzed the data to determine if data was collected continuously as designed or if there were substantial or frequent outages.

We define outages differently depending on the data type and sampling method. Accelerometer, motion, gyroscope, compass, and audio data can be collected with a constant sampling frequency throughout the whole session. For audio data, the maximum acceptable gap between data points is equivalent to 16 khz. For accelerometer, motion, gyroscope, and compass data, the maximum acceptable gap between data points is 0.02 s. An outage is any instance where the time between consecutive data points is larger than this predefined maximum acceptable time gap.

By default, the devices used in this study do not sample heart rate constantly and instead vary sampling rates based on detected activity. In order to maximize clinical utility, we designed MSMS to sample heart rate at a higher rate than these default settings. We defined a maximum acceptable time gap between consecutive collected data points based on tested device performance at this higher sampling frequency. An outage is any series of data where the time between consecutive data points is larger than this predefined maximum acceptable time gap.

MSMS collects the remaining data types (distance, steps, activity, and pedometer) only when the specific activity is sensed by the devices. Outages for these data types cannot be defined precisely because it would be impossible to differentiate between an outage and a period of no activity.

We defined data coverage as the ratio of the accumulated time between data points less than or equal to the respective maximum acceptable time gaps and the total amount of time in consideration. We calculated aggregations at the session level. A complete list of the data types collected sorted by device type is shown in Table 1, along with the data gap tolerances and if they included a corresponding time range or specific timestamp.

**Table 1:** List of data types collected via mobile devices and watches.

Device	Data type	Features	Data tolerance gap (s)
Mobile	Accelerometer	Acceleration vector	0.02
	Gyroscope	Device rotation across XYZ axes	0.02
	Compass	Magnetic field vector	0.02
	Motion	Attitude pitch, roll and yaw rotation rate on XYZ axes	0.02
		Gravity vector	
		User acceleration vector	
		Magnetic field vector	
	Audio	Waveform amplitude	1/sf
	Activity	Confidence of measurement and bool indicator of activity • Unknown activity • Stationary activity • Walking • Running • Cycling	Ad hoc sampling
	Pedometer	Number of steps walked	Ad hoc sampling
		Distance	
		Average active pace	
		Current pace	
		Current cadence	
		Ascended/ descended floors	
Both devices	Distance	Distance traveled in time window	Ad hoc sampling
	Steps	Number of steps walked in time window	Ad hoc sampling
Watch	Heart rate	Beats per minute estimate	10
	Watch accelerometer	Accelerometer vector	0.02
	Watch motion	Attitude pitch, roll and yaw	0.02
		Rotation rate on XYZ axes	
		Gravity vector	
		User acceleration vector	

### 2.5. Usability data

We collected data about the participant's and HCP's experience of using MSMS. HCPs presented participants with a usability survey after sessions 1, 4, and 8, to collect data about how well MSMS is tolerated and if it interferes with the therapeutic effects of Spravato sessions. HCPs then completed their own version of the usability survey. We calculated average scores and standard deviations to gain preliminary insight into whether participants and HCPs find MSMS acceptable to use during Spravato sessions.

## 3. Results

### 3.1. Participants

**Figure 2:**
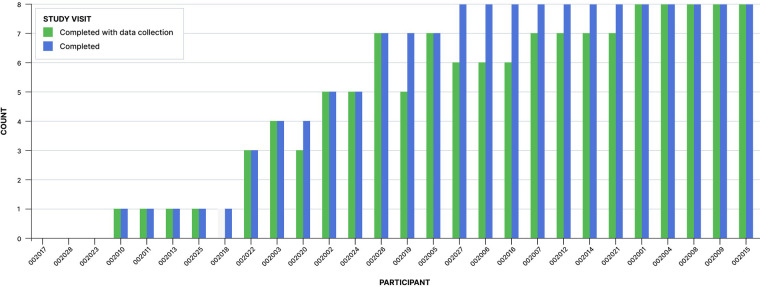
Number of Spravato sessions with or without MSMS data collection completed by each participant over the course of the study.

We collected data using MSMS between January 10, 2022, and June 22, 2022. A total of 28 participants consented to participate in the study. Consent did not obligate participants to undergo MSMS data collection for every Spravato treatment, they could choose to decline data collection for any or all Spravato sessions over the course of the study. Out of 28 consented participants, 24 participants underwent at least one study treatment session with MSMS data collection. Three participants did not schedule a Spravato session during the course of the study due to personal or clinical reasons, and one participant underwent one treatment session over the course of the study but MSMS was not used. Participants completed a total of 143 treatment sessions during the study, out of which 129 were conducted with MSMS. All sessions in this study were part of the participant's existing planned course of care; the modal number of treatment sessions with MSMS data collection was 7 with a range of 1–8. Figure 2 illustrates the distribution of scheduled sessions, completed sessions, and completed sessions with MSMS data collection. The overall sample was predominantly male (58%) and did not specify their race/ethnicity (60%). The mean participant age was 40 years old (SD = 15.5, Range 22–77). Participants generally exhibited mild to severe levels of depressive symptoms at the beginning of the study with an initial mean BDI score of 43.0 (±10.44) and an initial mean PHQ9 score of 13.9 (±6.2). Relatively high levels of depressive symptoms were not surprising for this study given that Spravato is indicated for TRD and MDD with suicidal ideation, and participants were only selected from a pool of subjects already receiving Spravato as a part of their current treatment plan.

### 3.2. Data quality

#### 3.2.1. Data collection in a clinical setting

MSMS successfully captured data in a real-world clinical setting during this study. MSMS also successfully transmitted from devices to our databases during every session where participants agreed to data collection. Sessions occurred in several locations throughout the study clinic where treatment was undertaken.

#### 3.2.2. Data corruption

We evaluated all collected data for quality in terms of the predefined ranges discussed in Section 2.4.2. Approximately 100% of every data type fell within these predefined ranges, indicating low to no data corruption, except for compass data. Only 81% of the compass data values were within the predefined range, the remaining data had extreme changes in a short period of time which were deemed unfeasible and indicated some sort of data corruption. We explain this further in the discussion section. Table 2 reports the full results of data within a realistic range per data type.

**Table 2:** Mean percentage of collected data within predefined ranges.

Device	Type	% in range
Mobile	Accelerometer	100.00
	Activity	100.00
	Compass	81.13
	Gyroscope	100.00
	Motion	99.94
	Pedometer	98.48
Both Devices	Steps	100.00
	Distance	100.00
Watch	Accelerometer	100.00
	Heart rate	100.00
	Motion	100.00

#### 3.2.3. Data coverage

Table 3 demonstrates the data coverage results for each data type. Information about the number of recordings per data type, mean, and standard deviation of coverage across sessions as well as the 25, 50, and 75th percentile of coverage value across the sessions (Q1, median, Q3) is displayed along with the minimum and maximum coverage per data type and session (min, max).

**Table 3:** Coverage statistics aggregated across sessions.

		Coverage (%)							
Device	Type	Mean	Std	Min	Q1	Median	Q3	Max	Number of records
Mobile	Accelerometer	98.20	3.52	82	98	100	100	100	129
	Audio	98.23	3.52	82	98	100	100	100	129
	Compass	98.20	3.52	82	98	100	100	100	129
	Gyroscope	98.20	3.52	82	98	100	100	100	129
	Motion	98.20	3.52	82	98	100	100	100	129
Watch	Accelerometer	94.77	17.46	0	96	100	100	100	129
	Heart Rate	92.63	17.59	0	93	98	100	100	129
	Motion	94.39	17.69	0	96	100	100	100	129

Phone data types such as accelerometer, audio, compass, gyroscope, and motion as well as watch accelerometer and motion data are collected constantly with a sampling period of 0.02 s. Analysis indicated that the phone data had approximately 98.2% mean coverage with very low variance. This means that on average, we were receiving data every 0.02 s or less for 98.2% of each session. Watch accelerometer, motion, and heart rate data indicated a lower average coverage rate of 94.8%, 94.3%, and 93% respectively with higher variance due to four outlying sessions where 0% of the session had adequate coverage by the smartwatch. These four sessions were for participants 002003, 002009, 002012, and 002021 and are explained further in the discussion section. The median coverage rate for the watch accelerometer and motion data was 100% and was 98% for heart rate.

**Figure 3:**
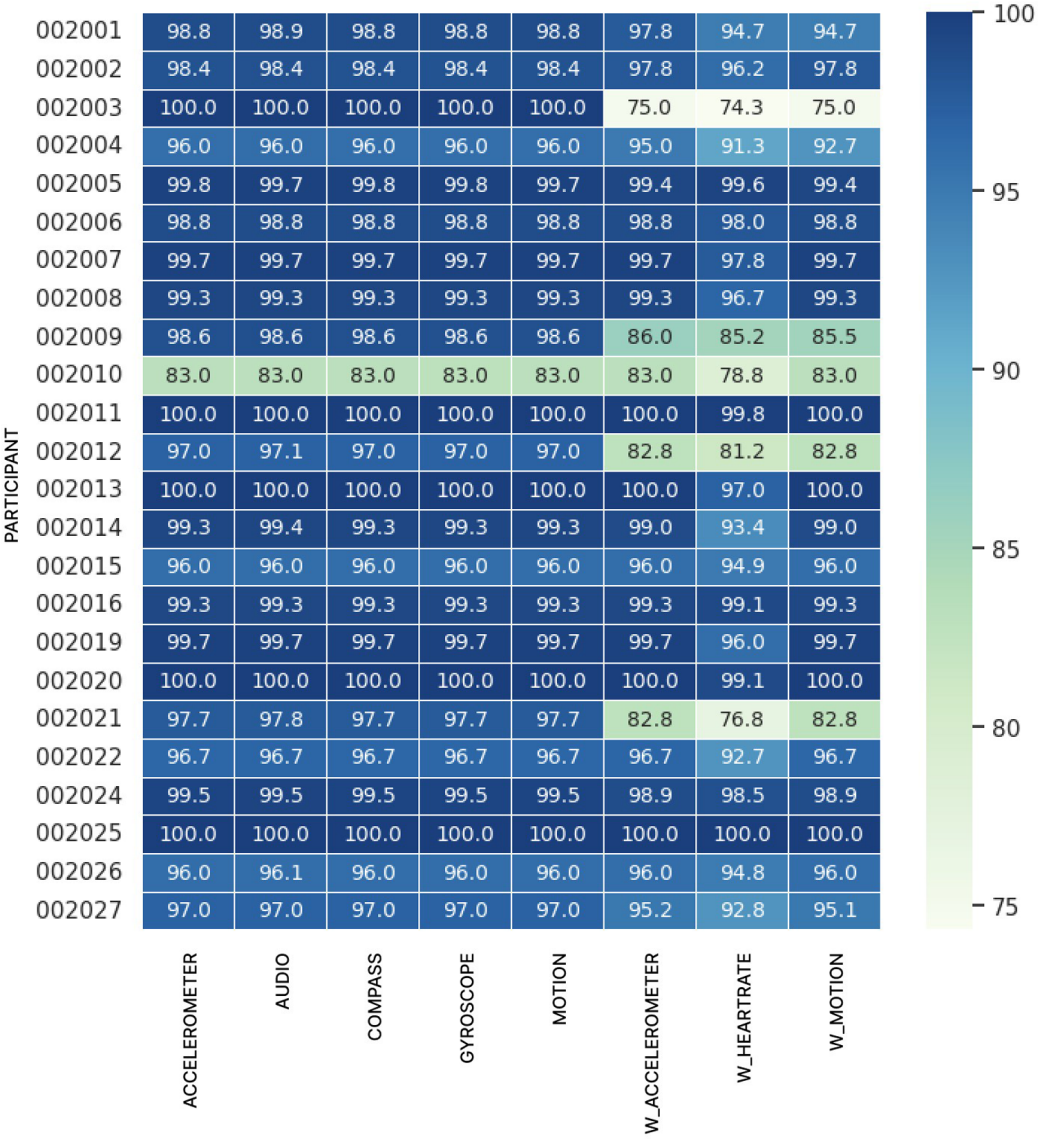
Mean data coverage (%) per participant with a colored heatmap.

Data coverages can be further broken down into participant-level views to observe whether we encounter any statistically significant abnormalities at this level of higher granularity. It's important to emphasize that not every participant has the same amount of data points collected (depending on how many sessions the participant undertook). Figure 3 presents the percentage mean coverage values over data types and users. The outlying lower coverage rates for Participant 002010 is explained in the discussion section.

### 3.4. Usability data

Participants and HCPs had the option to complete a usability survey after sessions 1, 4, and 8. These surveys aimed to collect data about how well MSMS is tolerated and if it interfered in any way with the therapeutic effects of Spravato sessions. Not all participants and HCPs chose to complete the survey each time it was offered. Additionally, participants who did not complete all eight sessions would not have been prompted all three times to answer the survey, i.e., a participant who completed seven sessions would only have the opportunity to answer the survey after sessions 1 and 4. Full copies of each usability survey are displayed in the Supplementary Materials section.

After sessions 1, 4, and 8, the surveys asked participants how aware they were of MSMS during their Spravato sessions. After sessions 4 and 8 only, the surveys asked participants how difficult it was to get used to using MSMS on a scale from 1 to 5. For both questions, a rating of 1 indicates the lowest value (“not at all aware”, “not at all difficult”) and 5 indicates the highest value (“very aware”, “very difficult”) Table 4 illustrates the results of this question across each session where the survey was given.

**Table 4:** Results from MSMS participant's usability survey on average across the course of the study, participants indicated low awareness and low difficulty getting used to MSMS™ during Spravato treatment.

	Session 1 awareness	Session 4 awareness	Session 8 awareness	Session 4 difficulty	Session 8 difficulty
Mean	1.9	1.7	1.8	1.1	1.0
Standard deviation	0.9	1.0	1.2	0.3	0.0
Total responses	22	15	10	15	10

The survey also asked participants after sessions 4 and 8 to answer “yes” or “no” to the question “After the last several weeks of use, do you think this session monitoring system has affected your therapy in any way?”. We received 26 total responses to this question, 100% of which answered “no”, indicating minimal to no effects on the therapeutic experience of Spravato.

HCPs also completed usability surveys to collect data on their experiences facilitating Spravato sessions with MSMS. The surveys asked HCPs to rate how confident they felt using MSMS and how time consuming the use of MSMS on a scale of 1–5, with 1 indicating the lowest value (not at all confident, not at all time consuming) and 5 indicating the highest value (extremely confident, extremely time consuming). Table 5 displays the results across each session these questions were asked.

**Table 5:** Results from the HCP's usability survey.

	Session 1 confidence	Session 4 confidence	Session 8 confidence	Session 1 time consuming	Session 4 time consuming	Session 8 time consuming
Mean	3.9	4.8	4.0	1.7	1.7	2.0
Standard deviation	1.3	0.4	1.4	0.5	0.5	1.4
# of respondents	7	6	2	7	6	2

10 unique HCPs responded to at least one survey. Average reported confidence using MSMS™ increased between sessions 1 and 4 and reported levels of time consuming were generally low throughout the course of the study.

## 4. Discussion

We conducted the first study of MSMS, a system being developed to monitor patients undergoing consciousness-altering experiences and provide relevant data and information to supervising health care providers. Consciousness-altering treatments are an emerging field of mental health care with extraordinary promise to alleviate symptoms from a variety of mental health disorders. This field is rapidly expanding, with many different compounds currently under development (37). The goal for the development of MSMS is to use passive monitoring to solve issues of scalability, access, and objectivity in this field to help administer these treatments safely and effectively. The present study aimed to determine the feasibility of MSMS by testing it in a clinical population undergoing Spravato treatment and examining the quality of data collected as well as the usability of the devices in the opinions of participants and HCPs.

Our primary aim for this study was to assess the feasibility of collecting passive data on individuals undergoing consciousness-altering treatments. FDA in their guidance “Software as a Medical Device (SAMD): Clinical Evaluation” requires that analytical or technical validation is performed as part of product life-cycle activities (38). Per this guidance, analytical validation measures the ability of a SaMD to accurately, reliably and precisely generate the intended technical output from the input data. Analytical or technical validation confirms and provides objective evidence that the software was correctly constructed—namely, SaMD correctly and reliably processes input data and generates output data with the appropriate level of accuracy, and repeatability and reproducibility (i.e., precision); and demonstrates that the software meets its specifications and that the software specifications conform to user needs and intended uses. By collecting data indicating high levels of data quality and usability in a clinical setting, this proof-of-concept study demonstrates the potential for devices such as MSMS to meet the above standards.

Our secondary aim for this study was to understand the quality of data that was collected during treatment sessions using MSMS. Approximately 100% of data collected during the study met criteria to be considered in a realistic range, indicating high fidelity across all data types. Data coverage was also excellent across data types, averaging above 92% per treatment session. Given the granularity of the sampling rates, the rates of coverage collected indicate a high degree of reliability in coverage across study treatment sessions. For example, heart rate data was programmed to be collected roughly every 10 s and had on average a 92.6% coverage rate. Throughout a two-hour treatment session, MSMS was able to collect on average approximately 667 separate samples of heart rate. As compared with the often intermittent or episodic manual collection of heart rate during typical Spravato sessions (18), this represents a substantial increase over the number of samples possible without the use of passive monitoring.

While this study sought to establish a baseline for the technical performance of MSMS, we also sought to better understand the subjective experience of using the system for both participants and HCPs facilitating treatment sessions. In addition to sufficient rates of technical performance, MSMS was well received by both participants and HCPs. Participants reported that MSMS was not burdensome, that they were generally unaware of it during treatment sessions, and that it did not interfere with the therapeutic benefits of their Spravato treatment. HCPs reported MSMS was not difficult or time consuming to set up, however they reported only medium levels of confidence with their own ability to appropriately use MSMS upon their initial session. However, by the fourth session in which they utilized the device, HCPs on average reported higher levels of confidence in their abilities, indicating that they were able to become adroit at integrating the device into their treatment sessions after only a small number of uses.

### 4.1. Data quality outliers

There are several outliers in the data that warrant further explanation. For data corruption rates, the only data type which had any substantially unrealistic values was compass data, where only 81% of collected values were within the range defined by our predefined ranges. We speculate that the drop in data fidelity may have been impacted by the clinical site's use of transcranial magnetic stimulation (TMS). The use of TMS was unrelated to the study but could have caused interference to the device's compass when they were in proximity to the large magnets used in TMS. Despite this drop in the fidelity of the compass data, MSMS demonstrated high overall data fidelity across various modalities.

During several sessions for participants 002003, 002009, 002012, 002021, the smartwatch, (which collected watch accelerometer, watch motion, and heart rate data) was in a lower sampling mode due to a technical error. During these sessions MSMS captured data, but the sampling frequency was too low and did not fall within the predefined range defined for reliable data coverage. We detected this error early in the study and sent an update to MSMS devices which fixed the problem for the remainder of the study. While it is important to consider any technical issues or limitations when determining feasibility, future updates to MSMS software will ameliorate such technical errors and further increase rates of coverage across data collection modalities.

As indicated in Figure 3, participant 002010 had lower coverage rates across both watch and phone data compared to other participants. Their coverage rates were skewed by one session which ended 28 min early, creating a substantial period of 0% coverage. This session ended early due to clinical reasons unrelated to this study.

### 4.2. Limitations

Overall, results from this study indicate that MSMS reliably and consistently captures multiple streams of data during a Spravato session, demonstrating the feasibility of passive monitoring using digital devices during consciousness-altering treatments. However, this study had several limitations that are important to consider. The sample size was small in terms of number of participants and HCPs who used MSMS as well as number of total sessions collected, which limits the generalizability of the results. This is especially notable in the usability surveys; for the session 8 survey there were only 10 participants (40% of total) and two HCPs (28% of total) who responded. One likely reason for this drop in response is that participants' Spravato sessions were scheduled based on their clinical need and not dictated by the study design. As a result, some participants did not have eight sessions within the study timeframe. Additionally, the surveys were offered to participants after treatment, so participants may have declined to complete them due to fatigue or other side effects from Spravato.

Another limitation is that the sensors and battery power in the devices used limited the granularity of data that could be collected. Devices for this study were chosen for practical purposes and do not represent the state of the art. Newer, more powerful devices could have enabled higher sampling rates or the collection of additional types of data.

Further, this study did not collect any data annotations. Annotations are labels applied to data points to help provide context for various data analyses. Collecting annotations from HCPs during a session or post-hoc via video recordings could have been helpful in improving our data analysis and enhancing our understanding of Spravato sessions. For example, our analysis of the movement data (distance, steps, activity, pedometer) was limited because it was impossible to distinguish between a period of no movement or a data outage. Data annotations could provide a way to confirm whether a participant's movement corresponds with the data we receive or if there is an issue with movement data collection. In addition to confirming the quality of data collection, annotations could help determine how the raw physiological data collected by MSMS corresponds to specific side effects of consciousness-altering drugs such as dizziness and disorientation. Movement data could also potentially serve as an indicator for clinical improvement considering its positive correlation with mental health (39, 40), although it is unclear if movement during the course of a consciousness-altering treatment session (typically less than 24 h) would indicate clinically meaningful data. Regardless, future research should involve annotation collection to further validate the abilities of MSMS to collect physiological data and better explore expanded monitoring capabilities. Despite these limitations, the promising results of this study help establish the feasibility of a product such as MSMS and the need for further research on passive monitoring during consciousness-altering treatment sessions.

## 5. Conclusion

Spravato and other consciousness-altering treatments show significant promise in treating a variety of mental health disorders, but using these substances as effective treatments comes with a significant patient monitoring burden. Passive monitoring could help alleviate this burden, potentially reducing the cost and increasing the accessibility of these treatments. We conducted the first study of the MindMed Session Monitoring System (MSMS), a passive monitoring system designed for consciousness-altering treatment sessions. Results indicated that the system reliably collected data during clinical treatment sessions with Spravato, with high rates of session coverage and low rates of data corruption. Additionally, user acceptability data indicated that participants and HCPs experienced a low burden while using the system and that it did not interfere with the therapeutic effects of a Spravato treatment session. More research and development are needed to fully explore the potential of passive monitoring systems such as MSMS.

## Data Availability

The original contributions presented in the study are included in the article/**Supplementary Material**, further inquiries can be directed to the corresponding author.
