# Supplementary material for: Evaluating passive physiological data collection during Spravato treatment
Source: Front Digit Health. 2023 Nov 29;5:1281529. doi: 10.3389/fdgth.2023.1281529 (PMC10716422; doi:10.3389/fdgth.2023.1281529)
Supplement: Supplementary file 1 [file Datasheet1.pdf]

Supplementary Material

1. Usability Surveys

Below are copies of the participant and HCP usability surveys administered for session 1, session 4, and session 8.

1.1 Session 1 Participant Usability Survey

1. What were your spontaneous thoughts after the clinician had presented the devices to you? [Free Text]
2. How much were you aware of having the devices on you during your treatment session? [1-5 Likert Scale]
3. How did you feel about wearing the devices during the treatment session? [Free Text]
4. What were your spontaneous thoughts after the clinician had taken the devices off you? [Free Text]
5. Do you normally wear other devices during the day on the wrist? [Yes or No]
6. Is there anything else you would like to tell us about your experience using the session monitoring system? [Yes or No]
7. If your previous answer is Yes, tell us what? [Free Text]

1.2 Session 4 Participant Usability Survey

1. How much were you aware of having the devices on your wrist during your prior treatment session over the last 2 weeks? [1-5 Likert Scale]
2. After the last 2 weeks of use, how easy or difficult was it to get used to wearing the devices during your treatment sessions? [1-5 Likert Scale]
3. How did you feel about wearing the devices during your prior treatment sessions over the last 2 weeks? [Free Text]
4. After the last 2 weeks of use, do you think this session monitoring system has affected your therapy in any way? [Yes or No]
5. If your previous answer is Yes, tell us how? [Free Text]
6. Is there anything else you would like to tell us about your experience using the session monitoring system? [Yes or No]
7. If your previous answer is Yes, tell us what? [Free Text]

1.3 Session 8 Participant Usability Survey

1. How much were you aware of having the devices on your wrist during your prior treatment session over the last several weeks? [1-5 Likert Scale]
2. After the last several weeks of use, how easy or difficult was it to get used to wearing the devices during your treatment sessions? [1-5 Likert Scale]
3. How did you feel about wearing the devices during your prior treatment sessions over the last several weeks? [Free Text]
4. After the last several weeks of use, do you think this session monitoring system has affected your therapy in any way? [Yes or No]
5. If your previous answer is Yes, tell us how? [Free Text]

6. What kinds of challenges might a patient face when engaging with this session monitoring system? [Free Text]
7. What would you need for a smooth integration of this session monitoring system in your therapy? [Free Text]
8. Is there anything else you would like to tell us about your experience using the session monitoring system? [Yes or No]
9. If your previous answer is Yes, tell us what? [Free Text]

1.4 Session 1 HCP Usability Survey

1. How confident did you feel using the MSMS devices for the first time? [1-5 Likert]
2. How time consuming do you find the MSMS device management to be? [1-5 Likert]
3. Would you need the support of a direct technical person during the first use of this product? [Yes/No]
4. Were there any specific challenges you encountered during your first device check & preparation? [Yes/No]
5. If your previous answer is yes, what were they? [Free Text]
6. Were there any specific challenges you encountered during your first device set-up on a patient? [Yes/No]
7. If your previous answer is yes, what were they? [Free Text]
8. Was there any specific information or knowledge you were missing prior to first use of the session monitoring system? [Yes/No]
9. If your previous answer is yes, what were they? [Free Text]
10. Have you experienced any specific issues during use of the session monitoring system? [Yes/No]
11. If your previous answer is yes, what were they? [Free Text]
12. Have you experienced any issues when taking devices off the patient & stopping the session? [Yes/No]
13. If your previous answer is yes, what were they? [Free Text]
14. If you could make any changes to the session monitoring system, what changes would you make? [Free Text]
15. Is there any other feedback you would like to provide us about the system or your experience using it? [Yes/No]
16. If your previous answer is yes, tell us what? [Free Text]

1.5 Session 4 HCP Usability Survey

1. How confident did you feel using the MSMS devices over the last 2 weeks? [1-5 Likert]
2. How time consuming did you find the MSMS device management to be over the last 2 weeks? [1-5 Likert]
3. After the last 2 weeks of use, would you need the support of a direct technical person during the use of this product? [Yes/No]
4. After the last 2 weeks of use, were there any recurring challenges you encountered during your device check & preparation? [Yes/No]
5. If your previous answer is yes, what were they? [Free Text]
6. After the last 2 weeks of use, were there any recurring challenges you encountered during device set-up on a patient? [Yes/No]
7. If your previous answer is yes, what were they? [Free Text]

8. During the last 2 weeks of use, have you experienced any specific issues during use of the session monitoring system? [Yes/No]
9. If your previous answer is yes, what were they? [Free Text]
10. During the last 2 weeks of use, have you experienced any issues when taking devices off the patient & stopping the session? [Yes/No]
11. If your previous answer is yes, what were they? [Free Text]
12. After the last 2 weeks of use, would you change anything on the MSMS product? [Free Text]
13. Is there anything else you would like to tell us about your experience using the session monitoring system? [Yes/No]
14. If your previous answer is yes, tell us what? [Free Text]

1.6 Session 8 HCP Usability Survey

1. How confident did you feel using the MSMS devices over the last several weeks? [1-5 Likert]
2. How time consuming do you find the MSMS device management now? [1-5 Likert]
3. After the last several weeks of use, would you need the support of a direct technical person during the use of this product? [Yes/No]
4. After the last several weeks of use, were there any (new) specific challenges you encountered during your device check & preparation? [Yes/No]
5. If your previous answer is yes, what were they? [Free Text]
6. After the last several weeks of use, were there any (new) specific challenges you encountered during device set-up on a patient? [Yes/No]
7. If your previous answer is yes, what were they? [Free Text]
8. During the last several weeks of use, have you experienced any (new) specific issues during use of the session monitoring system? [Yes/No]
9. If your previous answer is yes, what were they? [Free Text]
10. During the last several weeks of use, have you experienced any (new) issues when taking devices off the patient & stopping the session? [Yes/No]
11. If your previous answer is yes, what were they? [Free Text]
12. If you could make any changes in the product, what changes would you make now? [Free Text]
13. What kinds of challenges might clinician face when engaging with this session monitoring system? [Free Text]
14. What would you need at your workplace for a smooth integration of this session monitoring system in your clinical practice? [Free Text]
15. Is there anything else you would like to tell us about your experience using the session monitoring system? [Yes/No]
16. If your previous answer is yes, tell us what? [Free Text]

2. Supplementary Table 1

Supplementary Table 1. Predefined ranges used to determine realistic values and rates of change for data collected in this study. Data values or rates of change outside of these ranges are assumed to be inaccurate and corrupted due to device error.

**Device**	**Data Type**	**Acceptable Value**	**Acceptable Rates of Change**

	**Acceleration**	Absolute acceleration < 5g	Absolute rate of acceleration change < 50 ms^{-3} on a 0.02s interval
	**Gyroscope**	Absolute rotation rate on each axes < 7 rad/s	+/- 20 for a a wrist flick, delta between 2 reads
	**Compass**	Contains device biased magnetic field, unbiased field is controlled within motion sensor<br>motion magnetic field vector in <5, 120> microT	0</delta<360 degrees delta between 2 reads
**Phone**	**Motion**	Gravity sensor in <0.9g, 1.1g><br>absolute user acc (without gravity) < 3g<br>absolute rotation on each axes < pi rad<br>absolute rotation rate < 7 rad/s	Absolute rate of user acceleration change < 10 ms^{-3} on 0.02s interval
	**Audio**	Undefined	N/A
	**Distance**	Less than 200 steps per one sensor measurement<br>speed < 10kph	N/A
	**Steps**	Less than 200 steps per one sensor measurement<br>speed < 2 steps per second	N/A
	**Activity**	Check unique activity per timestamp	N/A
	**Pedometer**	Speed < 10kph<br>speed < 2 steps per second	N/A
	**Heart Rate**	HR beats per minute in <35, 190>	N/A
	**Watch Accelerometer**	Absolute acceleration < 5g	Absolute rate of acceleration change < 50 ms^{-3} on 0.02s interval
**Watch**	**Watch Motion**	Gravity sensor in <0.9g, 1.1g><br>Absolute user acc (without gravity) < 8g<br>Absolute rotation < pi rad<br>Absolute rotation rate < 30 rad/s	Absolute rate of user acceleration change < 40 ms^{-3} on 0.02s interval
